# Association of Lipid Profile and Body Mass Index with Periodontal Status in Patients with Dyslipidemia with and without Lipid-lowering Medication: A Cross-sectional Study

**DOI:** 10.3290/j.ohpd.b966783

**Published:** 2021-02-19

**Authors:** Suteera Techatanawat, Aurasri Komchornrit

**Affiliations:** a Lecturer, Department of General Dentistry, Faculty of Dentistry, Srinakharinwirot University, Bangkok, Thailand. Conceptualisation, investigation, data analysis, wrote original manuscript draft.; b Lecturer and Periodontist, Department of General Dentistry, Faculty of Dentistry, Srinakharinwirot University, Bangkok, Thailand. Conceptualisation, investigation, reviewed and edited manuscript.

**Keywords:** body mass index, dyslipidemia, high-density lipoprotein cholesterol, low-density lipoprotein cholesterol, periodontal disease

## Abstract

**Purpose:**

To investigate the relationship between periodontal parameters and lipid profiles.

**Subjects and Methods:**

A total of 48 subjects with dyslipidemia, consisting of 33 subjects who did not receive lipid-lowering medication (NLM) and 15 subjects who did receive lipid-lowering medication (LM) were enrolled in this cross-sectional study. Sixteen systemically healthy subjects were recruited as controls. The plaque index (PI), gingival index (GI), bleeding on probing (BOP), probing depth (PD), and clinical attachment level (CAL) were measured. The levels of triglyceride (TG), total cholesterol (TC), low-density lipoprotein cholesterol (LDL-C), and high-density lipoprotein cholesterol (HDL-C) levels were determined. The variables related to high cholesterol levels, including age, gender, waist circumference, and body mass index (BMI), were evaluated.

**Results:**

The LM group had a statistically significantly higher CAL in comparison with either the control or the NLM groups. TG was statistically significantly correlated with PD (ρ = 0.398, p = 0.001) and CAL (ρ = 0.349, p = 0.005). HDL-C was negatively correlated with PI (ρ = -0.371, p = 0.003), GI (ρ = -0.284, p = 0.025), and PD (ρ = -0.289, p = 0.023). The stepwise multiple regression analysis showed that BMI was statistically significantly associated with percentage of sites with BOP (β = 0.367, p = 0.003) and PD (β = 0.392, p = 0.002). CAL was statistically significantly influenced by age (β = 0.496, p < 0.001) and HDL-C (β = -0.259, p = 0.026).

**Conclusion:**

TG and HDL-C levels were correlated with periodontal status. BMI was found to be a stronger predictor of periodontal inflammation than serum lipid levels. No benefit of lipid-lowering medication on periodontal status was revealed.

Dyslipidemia is an abnormal metabolic condition which constitutes a major risk factor for atherosclerotic cardiovascular disease.^10^ It can present as a reduction of a high-density lipoprotein cholesterol (HDL-C) alone or in combination with either the elevation of plasma cholesterol or triglyceride (TG), leading to lipid peroxidation and systemic inflammation.^2^ Periodontal disease is an infectious disease of gram-negative bacteria and is regulated by cascades of inflammatory responses, which lead to periodontal tissue destruction. Both dyslipidemia and periodontitis share common characteristics, such as chronic inflammatory diseases with multifactorial aetiology. The bi-directional relationship between dyslipidemia and periodontal disease has been addressed in previous studies.^2,9^ The conditions of inflammation and infection can cause changes in lipid metabolism, such as the elevation of TG and reduced HDL-C levels.^7^ On the other hand, an impaired lipid metabolism also increases the severity of inflammation and infection by causing dysregulation of immune cells and the wound healing process.^2^

Statins are the first-line drugs used in the treatment of dyslipidemia. Besides the lipid-lowering effect, statins have been recognised to have pleiotropic properties, including anti-oxidative and anti-inflammatory effects.^21,25^ Thus, the potential benefits of statins in periodontal treatment have created a great deal of interest. Numerous studies reported better periodontal treatment results in subjects with periodontal disease who were taking either topically or systemically delivered statins.^11,17^ Sangwan et al^22^ reported statistically significantly lower mean probing depth (PD) and gingival index (GI) in dyslipidemia subjects using statins compared to those who did not take statins, which suggested the positive impact of statins on periodontal status. However, this remains a controversial, since another study reported similar average PD and GI of subjects using atorvastatin vs those who were not on the medication.^30^

The null hypothesis of this cross-sectional study was that the subjects with dyslipidemia who were taking lipid-lowering medication had less periodontal inflammation than those who did not take lipid-lowering medication. Therefore, this study aimed to compare the periodontal status of subjects with dyslipidemia, those who were and were not taking lipid-lowering medication, as well as systemically healthy controls. Secondly, the associations of serum lipid levels with periodontal parameters, including PD, clinical attachment level (CAL), and percentage of sites with bleeding on probing (BOP) were evaluated.

## Subjects and Methods

### Study Population

This cross-sectional study randomly recruited 48 subjects with dyslipidemia from the Srinakharinwirot Clinic (SWU Clinic), Bangkok, Thailand. The inclusion criteria included persons diagnosed with dyslipidemia for at least one year. The exclusion criteria were receiving periodontal treatment or antibiotic medication in the last six months, and a diagnosis of diabetes mellitus, thyroid disease, or kidney disease. Those subjects with a history of salivary gland pathology or radiotherapy in the head and neck area, fewer than 10 teeth, any clinical signs of intraoral inflammation or acute infection other than periodontal disease, a history of smoking and alcohol consumption, and pregnant or lactating women were also excluded. The control subjects consisted of sixteen systemically healthy subjects who came to the clinic for a routine medical check-up. The same exclusion criteria were used for the control subjects. Each subject understood the protocol of this research study and gave written informed consent. The study protocol was approved by the Institutional Human Research Ethics Committee, Srinakharinwirot University (SWUEC/F-060/2562).

### Clinical and Laboratory Examinations

The demographic data and medical history were recorded using a review of medical charts and a questionnaire. All subjects underwent blood collection for the measurement of fasting plasma glucose (FPG), total cholesterol (TC), TG, low-density lipoprotein cholesterol (LDL-C), and HDL-C. Venous blood samples were obtained from the antecubital vein after a 12-h fasting period. In this study, the cut-off values to identify dyslipidemia were set according to the guidelines of the Adult Treatment Panel III (ATP III) of the National Cholesterol Education Program, as follows: TC ≥ 200 mg/dL, TG ≥150 mg/dL, LDL-C ≥130 mg/dL, HDL-C < 40 in males or < 50 mg/dL in females.^1^

Full-mouth periodontal examinations (except the third molar) were performed by one examiner (AK [second author]). All teeth were probed at six different sites (mesio-buccal, mid-buccal, disto-buccal, mesio-lingual, mid-lingual and disto-lingual) using a UNC-15 periodontal probe (Hu-Friedy; Chicago, IL, USA). Plaque index (PI),^27^ PD, CAL, BOP, and GI^12^ were evaluated. Only the highest value of PD or CAL per tooth were used to calculate the average PD and CAL for each individual subject.^22^

### Statistical Analysis

The sample size was calculated using statistical power analysis software (G*power 3.1; Bonn, Germany). A power calculation indicated that when the sample size was 64, the test would have >90% power at an effect size of 0.50, assuming a significance level of 5%. The statistical analyses were performed using SPSS 25.0 software (SPSS; Chicago, IL, USA). The normality of all continuous data was analysed using the Shapiro-Wilk, and the chi-squared test was applied to analyse the categorical variables. The differences between groups were assessed using one-way ANOVA or the Kruskal-Wallis test, when appropriate. The post-hoc analyses were performed using the Games-Howell or Tukey’s test for normally distributed variables and the Mann-Whitney U-test for non-normally distributed variables. Spearman’s rank correlation (ρ) was applied to study the correlation between metabolic parameters and periodontal status. The association of lipid levels, covariate variables, and periodontal parameters (β) was analysed using stepwise multiple regression analysis. The dependent variables were mean PD, mean CAL, and percentage of sites with BOP, and factors such as age, gender, the body mass index (BMI), HDL-C, LDL-C, TG, and FPG were included in the model as independent variables. TC and waist circumference were not included in the regression model, since TC was highly correlated with LDL-C (ρ = 0.833, p < 0.001) and waist circumference was highly correlated with BMI (ρ = 0.831, p < 0.001). A two-sided p-value < 0.05 was considered statistically significant for all analyses.

## Results

### Characteristics of Subjects

The 64 subjects were randomly recruited, including 16 systemically healthy subjects (control group), 33 dyslipidemia subjects with no medication (NLM), and 15 dyslipidemia subjects receiving lipid-lowering medication (LM). The subjects in the LM group mainly received statins as lipid-lowering medication. There were no statistically significant differences in terms of gender, BMI, or exercise activity between the three groups. The subjects in the LM group were statistically significantly older and had a larger waist circumference than the other groups (Table 1).

**Table 1:** Demographic information of subjects in control, NLM, and LM group

Variables	Control group (n = 16)	NLM group (n = 33)	LM group (n = 15)	p-value
Age (year)	39.0^a^ (37.25, 46.0)	45.0 (36.5, 48.0)	56.0^a^ (36.0, 60.0)	0.018*
Gender (female)	14 (87.5%)	26 (78.8%)	9 (60.0%)	0.178^$^
Waist (cm)	87.0^b^ (78.0, 92.75)	87.0 (82.0, 95.5)	95.0^b^ (86.0, 101.0)	0.044*
BMI (kg/m^2^)	22.84 (20.14, 27.91)	24.03 (21.08, 26.25)	25.71 (23.67, 28.06)	0.127*
Lipid lowering medication	–	–	Statins: 13 (86.7%) Gemfibrozil: 2 (13.3%)	–
Exercise				
Yes No	10 (62.5%) 6 (37.5%)	21 (63.6%) 12 (36.4%)	5 (33.3%) 10 (66.7%)	0.123$

Bold denotes significant differences. Continuous data are presented as median (1st, 3rd quartile) and categorical data are presented as counts with percent values within brackets. * Kruskal-Wallis test; ^$^chi-squared test. Statistically significant difference between a-a, b-b at p < 0.05 according to the Mann-Whitney U-test. BMI: body mass index.

### Metabolic Status and Periodontal Parameters of Subjects

The NLM group had significantly higher levels of TC and LDL-C compared to the control and LM groups. The control group had a significantly lower TG level than the other groups. No statistically significant differences of FPG and HDL-C levels were found between the three groups (Table 2).

**Table 2:** Metabolic parameters and periodontal status of subjects in this study

Variables	Control group (n = 16)	NLM group (n = 33)	LM group (n = 15)	p-value
Fasting plasma glucose (mg/dL)	87.0 (82.25, 93.25)	89.0 (82.0, 95.0)	92.0 (88.0, 99.0)	0.128*
Total cholesterol (mg/dL)	182.0^a^ (174.25, 197.5)	233.0^a,b^ (207.0, 249.5)	184.0^b^ (169.0, 194.0)	**< 0.001** *
Triglyceride (mg/dL)	63.5^c,d^ (57.25, 85.5)	90.0^c^ (70.0, 149.5)	126.0^d^ (76.0, 172.0)	0.003*
HDL-C (mg/dL)	70.50 (58.75, 81.75)	63.0 (52.0, 78.0)	54.0 (43.0, 64.0)	0.053#
LDL-C (mg/dL)	102.0^e^ (95.75, 110.0)	136.0^e,f^ (125.0, 165.0)	121.0^f^ (103.0, 130.0)	< 0.001*
Number of teeth	27 24.0, 28.0)	27 (25.0, 27.5)	26 (20.0, 28.0)	0.318*
Plaque index (PI)	1.28 (1.05, 1.46)	1.30 (1.04, 1.49)	1.48 (1.13, 1.85)	0.082#
Gingival index (GI)	1.16 (0.62, 1.29)	1.08 (0.68, 1.42)	1.26 (0.92, 1.53)	0.224#
% sites with BOP	25.52 (9.04, 36.84)	24.11 (10.90, 41.88)	28.70 (15.18, 50.89)	0.659#
Probing depth (mm)	2.41^g^ (2.19, 2.73)	2.46^h^ (2.31, 2.81)	2.82^ g,h^ (2.50, 3.11)	0.013*
CAL (mm)	2.37^i,j^ (2.09, 2.63)	2.57^i,k^ (2.36, 2.87)	2.95^j,k^ (2.56, 3.75)	**0.001#**

Bold denotes significant differences. Data are presented as median (1st, 3rd quartile). * Kruskal-Wallis test; # one-way ANOVA. Significant difference between h-h at p < 0.05; c-c, d-d, f-f, g-g at p < 0.01; a-a, b-b, e-e at p < 0.001 according to the Mann-Whitney U-test. Significant difference between i-i, k-k at p < 0.05 and j-j at p < 0.01 with the use of Games-Howell test. HDL-C: high-density lipoprotein cholesterol, LDL-C: low-density lipoprotein cholesterol, CAL: clinical attachment level.

**Fig 1:**
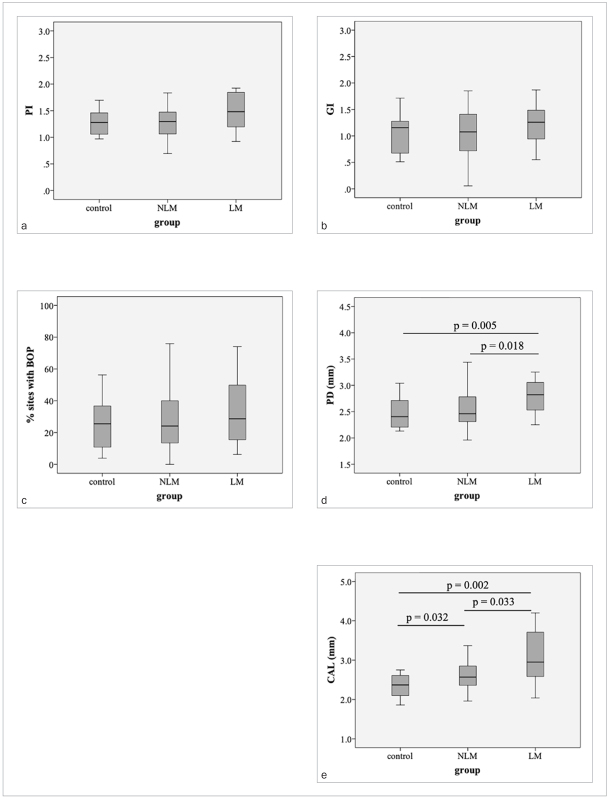
Periodontal status of subjects in control, NLM, and LM groups. a: plaque index (PI); b: gingival index (GI); c: % sites with bleeding on probing; d: probing depth (PD); e: clinical attachment level (CAL).

There were no statistically significant differences in terms of the number of teeth, PI, GI, and percentage of sites with BOP between the three groups (Table 2). Nevertheless, statistically significantly increased PD and CAL were shown in subjects with dyslipidemia. A statistically significant increase in PD was revealed in the LM group compared to both control and NLM groups (Fig 1d). Moreover, the LM group showed the highest mean CAL, followed by the NLM and the control groups (Fig 1e).

### Correlation Between Metabolic Parameters and Periodontal Status

The correlation between metabolic parameters and periodontal status was investigated using Spearman’s rank correlation (Table 3). BMI positively correlated with PI (ρ = 0.394, p = 0.001), GI (ρ = 0.339, p = 0.006), BOP (ρ = 0.285, p = 0.022), and PD (ρ = 0.288, p = 0.021). Waist circumference was also positively correlated with PI (ρ = 0.370, p = 0.003) and PD (ρ = 0.289, p = 0.02). No statistically significant correlation between FPG and any of the investigated periodontal parameters was found (Table 3).

**Table 3:** Correlations between age, BMI, waist, fasting plasma glucose, serum lipid levels, and periodontal parameters

Variables	Periodontal parameters				
	PI	GI	% Sites with BOP	PD	CAL
Age ρ p-value	-0.288** 0.021	-0.307* 0.014	-0.281* 0.025	-0.124 0.327	0.411** 0.001
BMI ρ p-value	0.394** 0.001	0.339** 0.006	0.285* 0.022	0.288* 0.021	-0.044 0.727
Waist ρ p-value	0.370** 0.003	0.229 0.069	0.191 0.131	0.289* 0.020	0.051 0.688
FPG ρ p-value	0.094 0.461	0.063 0.619	0.011 0.931	0.241 0.055	0.158 0.211
TC ρ p-value	-0.175 0.168	-0.018 0.887	0.088 0.488	0.028 0.828	0.036 0.776
TG ρ p-value	0.171 0.177	0.242 0.054	0.231 0.066	0.398** 0.001	0.349** 0.005
HDL-C ρ p-value	-0.371** 0.003	-0.284* 0.025	-0.237 0.063	-0.289* 0.023	-0.132 0.307
LDL-C ρ p-value	0.050 0.698	0.060 0.645	0.076 0.556	0.176 0.170	0.077 0.551

ρ: Spearman correlation coefficient. * Statistically significant correlation at p < 0.05; ** statistically significant correlation at p < 0.01. PI: plaque I GI: gingival index; BOP: bleeding on probing; PD: probing depth; CAL: clinical attachment level; BMI: body mass index; FPG: fasting plasma glucose; TC: total cholesterol; TG: triglyceride; HDL-C: high-density lipoprotein cholesterol; LDL-C: low-density lipoprotein cholesterol.

Moreover, cholesterol levels were correlated with periodontal status. The TG level was statistically significantly correlated with PD (ρ = 0.398, p = 0.001) and CAL (ρ = 0.349, p = 0.005). Conversely, HDL-C was negatively correlated with PI (ρ = -0.371, p = 0.003), GI (ρ = -0.284, p = 0.025), and PD (ρ = -0.289, p = 0.023) (Table 3).

### Association of Serum Lipid Levels and Covariate Variables with Periodontal Parameters

The results showed that BMI was statistically significantly associated with the percentage of sites with BOP (β = 0.367, p = 0.003) and PD (β = 0.392, p = 0.002). The subjects with high BMI seemed to have more gingival inflammation and a deeper PD. CAL was predicted by age (β = 0.496, p < 0.001) and HDL-C (β = -0.259, p = 0.026) (Table 4). The older subjects with lower HDL-C level tended to have more CAL.

**Table 4:** Association of serum lipid levels and covariate variables with periodontal status

Dependent variables	Model predictors	β	p-value	Adjusted R^2^	F
Percentage of sites with BOP	BMI	0.367	0.003	0.120	9.320
Average PD	BMI	0.392	0.002	0.140	10.897
Average CAL	age HDL-C	0.496 -0.259	< 0.001 0.026	0.239	10.604

Independent variables included in the model were age, sex, BMI, HDL-C, LDL-C, TG, and FPG. Only statistically significant variables are presented. BOP: bleeding on probing; PD: probing depth; CAL: clinical attachment level; HDL-C: high-density lipoprotein cholesterol; BMI: body mass index.

## Discussion

This cross-sectional study compared the periodontal status of subjects with dyslipidemia, including those taking and not taking lipid-lowering medication, as well as systemically healthy subjects. The relationship between serum lipid levels and periodontal status was also evaluated. The results showed that subjects with dyslipidemia who were taking lipid-lowering medication had a poorer periodontal status than those not taking the medication and healthy individuals. A statistically significant correlation between serum lipid levels and periodontal parameters was demonstrated. Interestingly, this study revealed that BMI was a stronger parameter than the serum lipid levels in terms of association with periodontal status.

The values of periodontal parameters, including CAL and PD, in subjects with dyslipidemia were higher than those of healthy individuals. The present study revealed the highest value of average CAL in the LM group, followed by those in the NLM and the control groups. Moreover, statistically significantly higher PD was also observed in the LM group compared to the others. These results imply a poorer periodontal health status in subjects with dyslipidemia, especially those in the LM group, in comparison to healthy individuals. This result is in line with previous studies, showing that subjects with dyslipidemia are more prone to periodontal disease. Fentoglu et al^3^ demonstrated statistically significantly increased PI, percentage of sites with BOP, PD, and CAL in subjects with dyslipidemia who were not on any lipid-lowering medications compared to those of normolipidemic individuals. Shivakumar et al^26^ compared periodontal status of subjects with mild (TC < 200 mg/dL, TG: 150-199 mg/dL, LDL-C: 100-129 mg/dL, HDL-C < 40-50 mg/dL), moderate (TC: 200-240 mg/dL, TG: 200-499 mg/dL, LDL-C: 130-159 mg/dL, HDL-C: 40-59 mg/dL), and severe dyslipidemia (TC>240 mg/dL, TG>500 mg/dL, LDL-C: 160-189 mg/dL, HDL-C < 40 mg/dL) with those of normolipidemic subjects. They indicated that PI, percentage of sites with BOP, PD, and CAL of the three groups with dyslipidemia were statistically significantly higher than those of the normolipidemic group. Moreover, the increasing trends of PI, BOP, PD, and CAL were demonstrated in the mild, moderate, and severe dyslipidemia groups, respectively.^26^ The possible explanation is that the impaired lipid metabolism could lead to the dysregulation of immune cells, including polymorphonuclear leukocytes (PMNs), which are crucial for the early immune response to periodontal infection.^2^ Moreover, serum lipids enhance the production of pro-inflammatory cytokines by PMNs and also inhibit macrophage production of several growth factors that are vital in the wound healing process.^2^ Therefore, individuals with dyslipidemia tend to have a poorer periodontal status.

The present study confirms the correlation between periodontal status and lipid profile, as the TG level was statistically significantly correlated with PD and CAL. The subjects with high TG tended to have deeper PD and increased CAL. This is comparable to other studies. Morita et al^14^ revealed that subjects with an elevated TG level (>149 mg/dL) had double the risk of periodontal disease. In addition, Sayar et al^24^ compared periodontal status among healthy subjects, subjects with hypertriglyceridemia who were on gemfibrozil, and those with hypertriglyceridemia who were not on medication. They found that the mean PD and CAL of the two groups of subjects with hypertriglyceridemia were statistically significantly higher than in healthy controls (p < 0.01). They also revealed statistically significantly positive correlations between TG and PD (r = 0.411, p < 0.001), TG and CAL (r = 0.355, p = 0.001), TC and PD (r = 0.359, p = 0.001) after controlling for age, BMI, group, and number of teeth. These results confirm the correlation between TG and periodontal status.

A trend of decreasing HDL-C levels was revealed in the LM and NLM groups compared to the control group, although these differences were not statistically significant (Table 2). Moreover, it was found that HDL-C was negatively correlated with PI, GI, and PD, and that subjects with low HDL-C seemed to have more plaque deposits and a poor periodontal status. This could be explained as a two-way relationship between dyslipidemia and periodontal status. Nepomuceno et al^15^ performed a meta-analysis of 19 cross-sectional studies in subjects with periodontitis but without any systemic disease, compared to periodontally healthy subjects. Their findings revealed that periodontal disease was significantly associated with the elevation of LDL-C and TG and reduction of HDL-C. Andriana et al^4^ investigated lipid profile and serum antibodies against various periodontal pathogens in subjects with untreated periodontitis and controls and demonstrated that serum antibodies (immunoglobulin G) against *P. gingivalis* and *A. actinomycetemcomitans* were statistically significantly associated with reduced HDL-C levels. This could imply the possibility of a bi-directional relationship between periodontal disease and dyslipidemia. Another possible explanation is oral hygiene behaviour. The two studies reported the association of proper oral hygiene care with a lower risk of metabolic syndrome, which encompasses the reduction of HDL-C and the elevation of TG levels as components.^8,19^ Thus, it is possible that subjects with high plaque deposits could increase the risk of dyslipidemia. Nevertheless, a further longitudinal study is required to confirm this hypothesis.

This study also revealed that BMI is a statistically significant parameter associated with periodontal status. BMI was statistically significantly correlated with PI, GI, and PD. Moreover, in the stepwise multiple regression analysis, statistically significant associations of BMI with a percentage of sites with BOP and PD were also demonstrated. The BMI is a conventional index of obesity and has been used in various studies regarding the relationship between overweight, obesity and periodontal disease. Wood et al^32^ showed a significant correlation between periodontitis and waist to hip ratio, BMI, fat-free mass, and subcutaneous fat. Saito et al^20^ also demonstrated that subjects with higher categories of BMI statistically significantly increased the adjusted risk of periodontitis compared to those with the lowest category of BMI. Moreover, a five-year cohort study of 3590 Japanese subjects revealed that the incidence of periodontitis (PD ≥ 4mm) was related to BMI in a dose-response relationship.^13^ In addition, BMI was also suggested as a predictive factor for non-surgical periodontal treatment outcome. Suvan et al^29^ studied a two-month outcome of non-surgical periodontal treatment in subjects with patients with normal weight, overweight, and obesity, all of whom were diagnosed with severe chronic periodontitis. They found that BMI (as continuous measures) was statistically significantly associated with higher mean PD and percentage of sites with PD >4 mm independent of age, smoking status, and dental plaque levels. In our study, BMI showed a stronger association with periodontal status than serum lipid levels, as the regression analysis revealed that only BMI that was statistically significantly associated with the mean PD and percentage sites of BOP. These results are similar to those of Saxlin et al,^23^ who demonstrated the association of high TG or low HDL-C levels with periodontal infection in obese subjects only. In addition, they found a strong association of deepened PD (PD ≥ 4mm) with BMI, despite adjusting for serum lipids. Thus, they confirmed the association of periodontal disease with BMI and suggested that there must be alternative mechanisms other than serum lipids that mediated the association. Nevertheless, the cross-sectional study by Sangwan et al^22^ demonstrated significant associations of TG level with GI, TC level with PD, and LDL-C level with CAL, despite the controlled confounding factors, including BMI. These contradictory results may be due to the difference in group of population, inclusion criteria, and study design. Further study in a larger population group with a design to eliminate the confounding factors should be performed to verify these contradictory results.

This study did not support a beneficial effect of statins on periodontal status. Statins are inhibitors of hydroxymethylglutaryl coenzyme A (HMG-CoA) reductase and are primarily used for controlling serum cholesterol levels. Previous studies suggested that statins could benefit periodontal wound healing due to their pleiotropic effects, including modulating inflammation, immune response, bone metabolism, and antibacterial effect.^17^ Lindy et al^11^ reported fewer periodontal lesions in statin users compared to non-statin users. Sangwan et al^22^ suggested the positive effect of statin in periodontal status, since they demonstrated that PD and GI were statistically significantly lower in patients with hyperlipidemia taking statin compared to those not taking statin. On the contrary, this study did not reveal the positive effects of statins on periodontal status. The LM group was mainly comprised of statin users (13 out of 15 subjects), who seemed to have worse periodontal status than the NLM and control groups, as they had statistically significantly higher PD and CAL than the other groups. A possible explanation could be confounding factors, including obesity. Although the subjects were randomly recruited, it still revealed that subjects with dyslipidemia who were taking lipid-lowering medication (LM group) tended to be obese (BMI ≥ 25 kg/m^2^)^5^ and had a statistically significantly larger waist circumference than did the other groups (Table 1). Periodontal disease has been proposed to be associated with obesity via the link of hyperinflammatory state.^28^ Adipose tissue is an active endocrine organ that secretes various adipokines that regulate lipid metabolism, glucose and vascular homeostasis.^6^ As a result of obesity, a low-grade systemic inflammation may occur due to increased production of adipokines, including pro-inflammatory cytokines and chemokines by adipose tissue cells.^18^ Several adipokines, including interleukin-6, tumor necrosis factor-a, adiponectin, leptin, and resistin have been reported to be associated with periodontal disease.^28^ For example, adiponectin, which is found at reduced levels among obese people, was suggested to enhance periodontal tissue wound healing. Its expression can be inhibited by *P. gingivalis*, which is a major periodontal pathogen.^16^ Thanakun et al^31^ reported increased dental inflammation and periodontal diseases in overweight and obese Thai subjects compared to those of a normal weight. They also revealed that obesity was positively associated with leptin and C-reactive protein levels and negatively associated with adiponectin levels. Collectively, it is possible that the subjects in the LM group were more prone to poor periodontal status due to obesity. Moreover, in order to use statins to benefit periodontal health, local delivery is suggested to be more effective than oral intake, as high doses are required for oral intake to achieve optimum effects on periodontal tissue. However, high doses can cause various side effects, including nephrotoxicity, hepatotoxicity, and xerostomia.^17^

The findings in the present study should be interpreted with caution. First, the causal relationship between lipid profile, BMI, and periodontal status cannot be established due to the limitations of a cross-sectional study. Second, this study did not recruit subjects in the LM group with different ranges of serum lipid levels; thus, this may affect the periodontal status of the LM group and obscure the beneficial effect of statin intake on periodontal status. A further prospective cohort study, recruiting periodontally healthy subjects with different lipid profiles and BMI ranges, and measuring the significant pro-inflammatory cytokines should be encouraged for a better understanding of the underlying biological mechanisms.

## Conclusion

This study confirms the association of serum lipid levels with periodontal status. However, the results revealed that subjects with dyslipidemia who were taking lipid-lowering medication had a poorer periodontal status than those who were not taking the medication and healthy individuals. Therefore, the benefit of statin intake with regard to periodontal status was not shown in this study. Moreover, this study suggests BMI as a stronger parameter than serum lipid levels for predicting periodontal inflammation.
